# A cross-continental comparative analysis of the neurological manifestations of Long COVID

**DOI:** 10.3389/fnhum.2025.1760173

**Published:** 2026-01-28

**Authors:** Millenia Jimenez, Melissa Lopez, Janet Miller, Njideka U. Okubadejo, Carolina Hurtado, Anurag Kumar Singh, Oluwadamilola O. Ojo, Diego F. Rojas-Gualdron, Iorhen Akase, Osigwe P. Agabi, Kamlesh Kumar, Balvir Singh Tomar, Deepak Nathiya, Rebecca Jules, Eric M. Liotta, Igor J. Koralnik

**Affiliations:** 1 The Ken and Ruth Davee Department of Neurology, Northwestern University Feinberg School of Medicine, Chicago, IL, United States; 2 Faculty of Clinical Sciences, Department of Medicine, College of Medicine, University of Lagos, Lagos, Nigeria; 3 Department of Medicine, Lagos University Teaching Hospital, Lagos, Nigeria; 4 School of Medicine, CES University, Medellin, Colombia; 5 Department of Pharmacy Practice, National Institute of Medical Science and Research, Jaipur, India; 6 Department of Neurology, National Institute of Medical Sciences and Research, Jaipur, India; 7 Institute of Pediatric Gastroenterology and Hepatology, National Institute of Medical Sciences and Research, Jaipur, India; 8 Global Neurology Program, Havey Institute for Global Health, Northwestern University, Chicago, IL, United States

**Keywords:** global neurology, Long COVID, neurology, PASC, post acute sequelae of SARS-CoV-2 infection, SARS-CoV-2

## Abstract

**Objective:**

To compare demographics, comorbidities, neurologic symptoms, quality of life, and cognitive outcomes among adult individuals with neurologic manifestations of post-acute sequelae of SARS-CoV-2 infection (Neuro-PASC) across countries with varying income levels: the United States (U.S.), Colombia, Nigeria, and India.

**Methods:**

In this multi-country observational study, participants were evaluated in hospital clinics and recruited from institutional databases between 2020 and 2025. Patients were categorized as post-hospitalization Neuro-PASC (PNP) or non-hospitalized Neuro-PASC (NNP). Cognitive assessments were performed using the NIH Toolbox (U.S. and Colombia), the Montreal Cognitive Assessment (Nigeria), or the Mini-Mental State Examination (India).

**Results:**

A total of 3,157 participants were enrolled (652 PNP; 2,505 NNP). PNP patients were predominantly male except in the US, while NNP patients were predominantly female, except in India. The most frequent neurologic symptoms were brain fog, myalgia, dizziness, headache, and sensory disturbances, with frequency highest in the U.S. and lowest in India. There were significant differences for most neurologic and non-neurologic symptoms of PASC, driven by higher frequencies in U.S. and Colombia in both PNP and NNP cohorts. In addition, cognitive impairment measured with different instruments varied across countries for both PNP and NNP groups. Multiple correspondence analysis showed clustering of symptom burden between U.S./Colombia and Nigeria/India.

**Conclusion:**

Neuro-PASC presents globally but symptom burden, and psychological distress vary across regions, likely influenced by sociocultural factors, healthcare access, and diagnostic tools. These findings highlight the need for culturally-adapted screening and post-COVID care worldwide.

## Introduction

As of December 2025, more than 778 million cases of COVID-19 have been reported worldwide, with 7.1 million deaths (World Health Organization, n.d.). Many survivors of COVID-19 develop persistent symptoms, known as Long COVID or post-acute sequelae of SARS-COV-2 infection (PASC). This syndrome is defined as a continuous, relapsing and remitting, or progressive disease state that is present for at least 3 months after SARS-CoV-2 infection and affects one or more organ systems (National Academies of Sciences, Engineering, and Medicine et al., 2024). It has been estimated that more than 400 million people worldwide suffer from Long COVID, resulting in a detrimental annual economic impact of $1 trillion, equivalent to ~1% of the global economy (Al-Aly et al., 2024). Published studies (including from this group) have shown the importance of neurologic manifestations of PASC (Neuro-PASC) which are the most frequent cause for consultation to our post-COVID clinic (Bailey et al., 2023; Crivelli et al., 2022). In addition, we have demonstrated that these vary based on the severity of the precedent acute COVID-19 (Perez Giraldo et al., 2023). Several studies have examined the global impact of Long COVID (Ramonfaur et al., 2024; Global Burden of Disease Long CC et al., 2022; Elboraay et al., 2025; Chen et al., 2022; Pazukhina et al., 2024; Seighali et al., 2024), however, those studies have not directly compared neurological manifestations of PASC using primary patient data across diverse economic and cultural settings, and therefore whether Neuro-PASC affects people differently in various geographic areas remains a knowledge gap.

To characterize this condition, we conducted separate collaborative studies in four countries worldwide, focusing on Neuro-PASC. The independent results have been previously published for the US (Perez Giraldo et al., 2023; Mukherjee et al., 2025; Choudhury et al., 2024), Nigeria (Akase et al., 2024), Colombia (Hurtado et al., 2024), and India (Singh et al., 2025). This allowed us to identify patterns in the frequency and characteristics of symptoms in these geographical locations.

The primary aim of this study was to perform a cross-continental comparative analysis of the demographics, comorbidities, signs and symptoms, subjective quality of life and cognitive function of individuals with Neuro-PASC in the United States (U.S.), Colombia, Nigeria, and India. The secondary aim was to explore associations based on the country’s economic status (The World Bank, n.d.).

## Methods

### Ethics and approvals

This report follows the STROBE statement for cohort studies (von Elm et al., 2008). This study was a collaboration between four university-affiliated hospitals or research institutions in the U.S. (North America), Colombia (South America), Nigeria (Africa), and India (Asia). Institutional Review Board (IRB) approval was obtained locally at each site as well as through the coordinating institution in the U.S. (Northwestern University, Chicago, IL): Chicago, United States (Northwestern University IRB STU00212583), Medellín, Colombia (CES University IRB code 1099, session 214 of 2022), Lagos, Nigeria (Lagos University Teaching Hospital IRB), and Jaipur, India (National Institute of Medical Sciences and Research IEC/P- 836/2024).

### Study design and setting

This was a multi-site secondary data analysis of four observational studies conducted in geographically and socioeconomically distinct regions: Chicago (U.S.), Medellin (Colombia), Lagos (Nigeria), and Jaipur (India). All studies were designed around a common protocol developed at the Northwestern Memorial Hospital’s (NMH) Neuro-COVID-19 clinic in Chicago, United States. Participants were evaluated in person or via telehealth. Data collected included demographic characteristics, COVID-19 status (confirmed by SARS-CoV-2 PCR, rapid antigen, or serology), comorbidities (self-reported and/or extracted from electronic medical records), and persistent neurological symptoms consistent with Long COVID (National Academies of Sciences, Engineering, and Medicine et al., 2024). Subjective measures of depression and anxiety were collected in the U.S., Colombia, and India. Objective cognitive assessments were conducted across all four sites. The study compared two clinical groups at each location: post-hospitalization Neuro-PASC (PNP) and non-hospitalized Neuro-PASC (NNP) patients.

### Participants

All study sites included adult individuals ≥18 years of age.

#### United States

All participants who presented with lingering neurologic symptoms lasting 3 months from documented SARS-CoV-2 infection were evaluated at the Neuro-COVID-19 clinic of Northwestern Memorial Hospital in Chicago, following referral by their primary care providers or self-referral via a hospital-operated COVID-19 hotline. Participants were assessed in person or remotely via telehealth, in compliance with U.S. federal telemedicine policies under the CARES Act between 5/13/2020 and 2/7/2025. Investigations of smaller subgroups from this population had been reported previously (Perez Giraldo et al., 2023; Mukherjee et al., 2025; Choudhury et al., 2024).

#### Colombia

Patients diagnosed with COVID-19 between April 2023 and August 2023 were recruited from the CES Clinic (previously hospitalized) and through IRB-approved social media outreach (non-hospitalized community members) between April 2023 and December 2023 as previously described (Hurtado et al., 2024).

#### Nigeria

Participants were recruited from the institutional COVID-19 database at Lagos University Teaching Hospital (LUTH) between April 1, 2020, and December 1, 2022, as previously described (Akase et al., 2024).

#### India

Participants were identified from the NIMS University Electronic Medical Record database for the period of March 1st, 2020, to March 31st, 2023 as previously described (Singh et al., 2025). Both post-hospitalization and non-hospitalized patients were contacted and assessed either in person or via telephone.

### Variables and measurements

Neurologic symptoms and signs attributed to Long COVID, determined by neurological examination, were defined as main outcome variable. Brain fog was defined uniformly as problems with concentration, memory and thinking occurring since COVID-19. Other Long COVID symptoms (fatigue depression/anxiety, Shortness of breath, insomnia, chest pain, dysautonomia, gastrointestinal symptoms) were also explored. As secondary outcome’s neurocognitive function and patient reported outcomes (PROMIS) were considered. Validated psychometric tests were employed according to availability in each site, as presented in Table 1; except for Nigeria where quality-of-life measures were not included. Additional variables were included to characterize history of COVID-19, premorbid comorbidities, and other relevant sociodemographic characteristics. Participants were categorized into post-hospitalization neuro-PASC (PNP) and non-hospitalized neuro-PASC (NNP) groups based on hospitalization status at the time of acute COVID-19.

**Table 1:** Instruments used to evaluate cognitive function, depression and anxiety in Neuro-PASC patients in the United States, Colombia, Nigeria and India.

	USA	Colombia	Nigeria	India
Evaluation of cognitive function	NIH Toolbox	NIH Toolbox	MoCa	MMSE
Evaluation of depression and anxiety	PROMIS	PROMIS	N/A	DASS-21

NIH, National Institutes of Health; PROMIS, Patient Reported Outcomes Information System; MoCa: Montreal Cognitive assessment; MMSE, Mini Mental State Examination; DASS-21, Depression Anxiety and Stress scale-21; N/A, not available.

NIH Toolbox 2.1 (Gershon et al., 2010; Weintraub et al., 2013; Gershon et al., 2013) and PROMIS English and Spanish versions were employed in United States and Colombia (Lai et al., 2014; Lai et al., 2011), the Identification and Intervention for Dementia in Elderly Africans (IDEA) test (Paddick et al., 2015) and the Montreal Cognitive Assessment (MoCA) test (Nasreddine et al., 2005) were employed in Nigeria, and the Mini-Mental State Examination (MMSE) (Folstein et al., 1975) was employed in India.

Neurological examination and cognitive testing were performed by neurologists, assisted by neurology fellows or physicians assistants.

### Bias

To control for information bias, neurological examinations cognitive testing and clinical assessments were conducted by neurologist, neurology fellows and physician assistants. Data collection was performed in a prespecified database with variable dictionary. All data were securely entered into an encrypted REDCap database. Research study coordinators performed data curation and validation under the supervision of the principal investigator. To control for selection, bias the fulfillment of eligibility criteria among potentially eligible patients was thoughtfully assessed during neurological examination.

### Quantitative variables

#### Cognitive function

Cognitive function was assessed using different, locally-validated tools: the National Institute of Health (NIH) Toolbox (version 2.1) was used in the U.S. and Colombia to evaluate cognitive function. It includes assessments of processing speed (pattern comparison processing speed test), attention (flanker inhibitory control and attention test), executive function (dimensional change card-sorting test), and working memory (list-sorting working memory test). Each subtest yields a T-score, which was combined into a composite cognitive T-score. A T-score of 50 reflects the normative U.S. reference population, with a standard deviation (SD) of 10. Scores below one SD (<40) were considered indicative of cognitive impairment. The NIH Toolbox scores were additionally standardized across age, sex, education, race, and ethnicity (Gershon et al., 2010; Weintraub et al., 2013; Gershon et al., 2013). In Nigeria, the Montreal Cognitive Assessment (MoCA) was used to screen for cognitive impairment. A MoCA score below 26 was considered abnormal. In India, the Mini-Mental State Examination (MMSE), a 30-point screening tool, was used to assess orientation, memory, attention, language, and visuospatial skills. Scores of 24 or higher were considered normal, while lower scores suggested varying degrees of cognitive dysfunction.

#### Depression and anxiety

In the U.S. and Colombia, patient-reported outcomes were collected using the PROMIS Quality of Life (QoL) scales. PROMIS scores are standardized T-scores with a mean of 50 and SD of 10. Scores above 55 were considered indicative of abnormal psychological burden, reflecting elevated symptoms of depression and anxiety (Lai et al., 2011, 2014). In Nigeria, patient-reported outcome measures for depression or anxiety were not collected. In India, psychological distress was assessed using the Depression, Anxiety, and Stress Scales (DASS-21), a 21-item self-report questionnaire covering three subscales. This study focused on the depression and anxiety subscales. Each item is rated on a 4-point Likert scale (0 = Did not apply to me at all; 3 = Applied to me very much or most of the time). The scores for each subscale are summed and then multiplied by two to match the DASS-42 scoring system - normal (0–9), mild (10-13), moderate (14-20), severe (20-27), extremely severe (28+), extremely Severe (28+). A composite score was calculated to determine abnormal levels of depression and anxiety.

#### Multiple correspondence analysis

To summarize and visualize the multidimensional symptom profiles of the PNP and NNP cohorts and the relationships between the Neuro-PASC symptoms, we performed multiple correspondence analysis (MCA) using those symptoms reported as present in ≥ 10% of patients in the overall cohort. MCA results are presented graphically as patient and symptom point clouds in two-dimensional space, defined by the first and second principal component dimensions (the two orthogonal axes with the largest portion of the data inertia, or amount of variation, explained by the component). In the MCA graphs, points further from the origin have a greater influence on the component axes. Patients plotted in similar locations in space tend to have similar symptom profiles, and symptom categories with similar patient profiles are grouped together. MCA was performed using the FactoMineR package in R (R version 4.2.1, Vienna, Austria). We used the Kruskal-Wallis rank sum test to identify if countries differed by their MCA principal components.

For MCA principal components that differed between countries, we performed multiple linear regression to determine if the association between the MCA component and country was independent of patient age and sex.

### Statistical methods

Descriptive statistics were used to summarize the following: number of patients (frequency), mean (standard deviation), for normally distributed variables and median (interquartile range [IQR]) for non-normally distributed variables. Categorical data such as comparisons of sex, race/ethnicity, frequency of signs and symptoms, visit types, and pre-existing comorbidities were reported as counts and percentages, while continuous data were presented as means and standard deviations (SD) for normally distributed data, or medians and interquartile ranges (IQR) for non-normally distributed data. Comparative analyses between regions were conducted using Fisher’s Exact Test and Chi-Squared Test for categorical variables. For continuous variables, one-way analysis of variance (ANOVA) was used for normally distributed data, and the Kruskal-Wallis test for non-normally distributed variables. Given particularly interesting differences in the occurrence of brain fog and depression/anxiety between countries, we performed binary logistic regression to determine if the association between occurrence of these symptoms and country was independent of patient age and sex.

Measure of cognition and psychological distress scores were analyzed for PROMIS and NIH Toolbox measures (used in the U.S. and Colombia), and composite T-scores were calculated by averaging subtest T-scores. Scores were then categorized as normal or abnormal based on established standardized test cutoffs. In India and Nigeria, where cognitive assessments were conducted using the MMSE and MoCA, respectively, the single total scores provided by each tool were used to classify participants as cognitively impaired or not, according to standard test cutoffs.

Psychological measures of depression and anxiety were performed using PROMIS (U.S., Colombia) and DASS-21 (India), with classification into normal and abnormal categories based on established scoring thresholds.

With the exception of MCA and regression models, which were performed in R (R version 4.2.1, Vienna, Austria), all statistical analyses were performed using GraphPad Prism version 10.0.2. Study data was collected and managed using the REDCap electronic data capture platform. To support interpretation, cognitive function across regions was summarized and visualized using the relevant standardized tools (NIH Toolbox, MoCA, MMSE). Subjective psychological measures were also summarized and visualized by region, using PROMIS (U.S., Colombia) and DASS-21 (India), with classification into normal and abnormal categories based on established scoring thresholds.

## Results

### Participants

**Figure 1:**
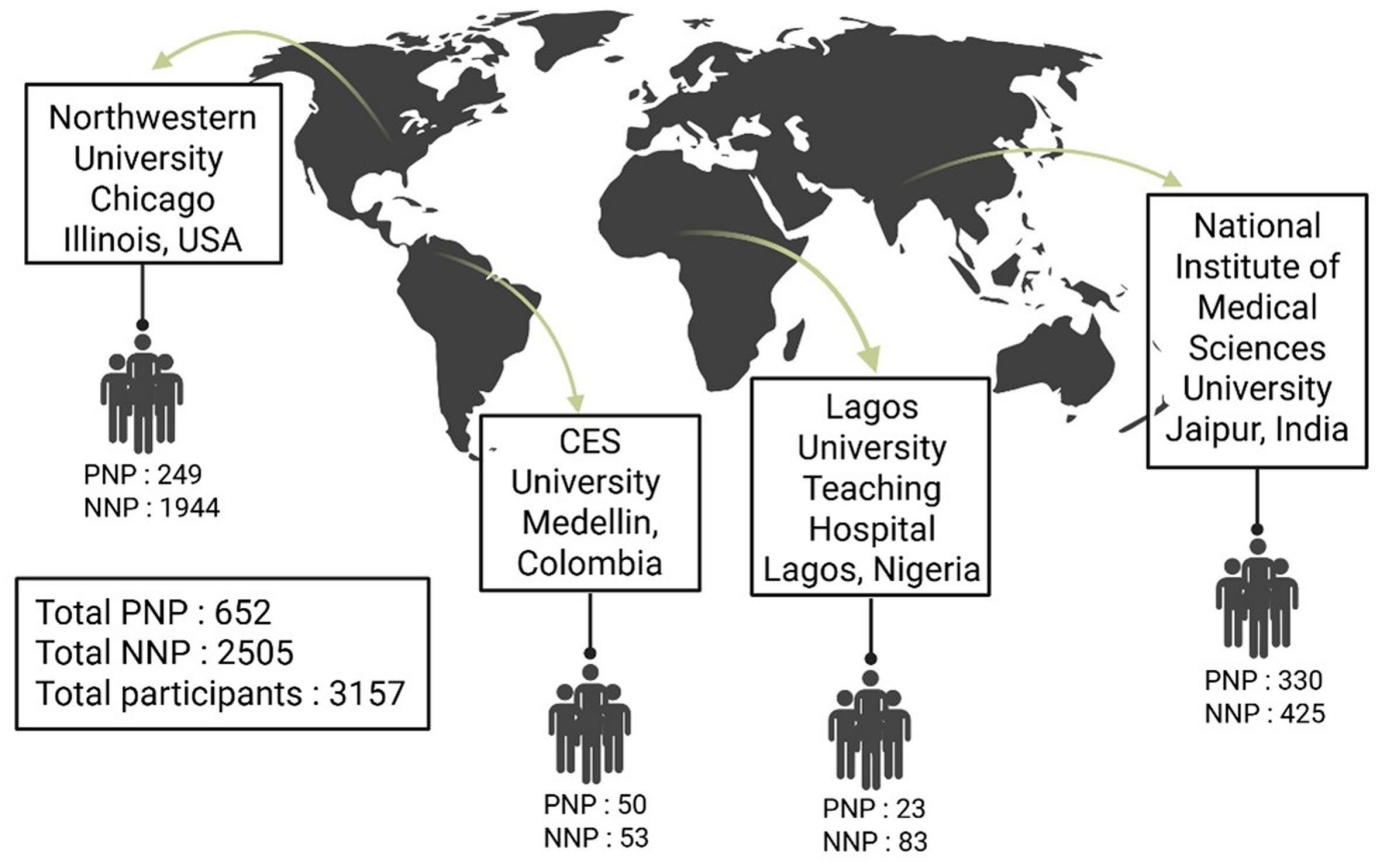
World map showing graphical distribution and numbers of study participants with neurological manifestations of post-acute sequelae of SARS-CoV-2 infection (Neuro-PASC) belonging to two groups: post-hospitalization Neuro-PASC (PNP) and non-hospitalized Neuro-PASC patients. Collaborative sites span across the United States, Colombia, Nigeria, and India.

Figure 1 portrays a world map illustrating the breakdown of the study population and their global distribution. There were a total of 3,157 participants (652 PNP and 2,505 NNP patients) from four geographic areas: 249 PNP and 1944 NNP from the United States; 50 PNP and 53 NNP from Medellin, Colombia; 23 PNP and 83 NNP from Lagos, Nigeria; and 330 PNP and 425 NNP from Jaipur, India.

### Descriptive data

#### Patients’ demographics and medical history

Table 2 shows demographic data by history of hospitalization for COVID-19 and geographic location. Of the 652 PNP patients who participated in this study, the mean age was 48.3 years old, while the 2,505 NNP patients had a mean age of 46.2 years with significant differences between the four groups (*p* < 0.0001). Overall, there was a predominance of males among PNP patients (52.5%) with significant differences among the four groups (*p* = 0.0009), driven by a higher percentage of females in USA only. Conversely, there was a predominance of females in the NNP group overall (62.5%) with significant differences between the four group (*p* < 0.0001) driven by a higher percentage of males in India only. By design, ethnic composition differed significantly between groups (*p* < 0.0001) All 3,157 participants had confirmed SARS-CoV-2 infection with either PCR, serological, or antigen testing. More than 80% of participants in the USA, Colombia and India had received SARS-CoV-2 vaccination, but vaccination status in Nigeria was not available.

**Table 2:** Demographics in post-hospitalization and non-hospitalized Neuro-PASC patients in the United States, Colombia, Nigeria, and India.

	Overall PNP	USA PNP	Colombia PNP	Nigeria PNP	India PNP	*p-*value	Overall NNP	USA NNP	Colombia NNP	Nigeria NNP	India NNP	*p-*value
Population total (*n*)	652	249	50	23	330		2505	1944	53	83	425	
Age, years (mean (1SD))	48.3 (16.3)	57 (13.6)	50.8 (10.7)	49.2 (12.7)	41.1 (15.6)	**<0.0001**	46.2 (26.3)	48.2 (28.5)	37.6 (12.2)	41.2 (12)	39.3 (15.4)	**<0.0001**
Gender, *n* (%)						**0.0009**						**<0.0001**
Male	342 (52.5)	108 (43.4)	26 (52)	17 (73.9)	191 (57.9)		939 (37.5)	651 (33.5)	13 (24.5)	41 (49.4)	234 (55)	
Female	310 (47.5)	141 (56.6)	24 (48)	6 (26.1)	139 (42.1)		1566 (62.5)	1293 (66.5)	40 (75.5)	42 (50.6)	191 (44.9)	
Ethic composition, *n* (%)						**<0.0001**						**<0.0001**
White	140 (21.5)	140 (56.2)	0 (0)	0 (0)	0 (0)		1395 (55.7)	1394 (71.1)	1 (1.9)	0 (0)	0 (0)	
Black	72 (11)	49 (16.7)	0 (0)	23 (100)	0 (0)		217 (8.7)	134 (6.9)	0 (0)	83 (100)	0 (0)	
Asian	337 (51.7)	7 (2.8)	0 (0)	0 (0)	330 (100)		489 (19.5)	64 (3.3)	0 (0)	0 (0)	425 (100)	
Hispanic	86 (13.2)	36 (14.5)	50 (100)	0 (0)	0 (0)		211 (8.4)	159 (8.2)	52 (98.1)	0 (0)	0 (0)	
Other	10 (1.5)	10 (4)	0 (0)	0 (0)	0 (0)		69 (2.8)	69 (3.5)	0 (0)	0 (0)	0 (0)	
Not specified	7 (1.1)	7 (2.8)	0 (0)	0 (0)	0 (0)		125 (5)	124 (6.4)	0 (0)	0 (0)	0 (0)	
Type of SARS-CoV-2 testing, *n* (%)						**<0.0001**						**<0.0001**
PCR	508 (77.9)	123 (49.4)	32 (54)	23 (100)	330 (100)		1538 (61.4)	998 (51.3)	32 (60.4)	83 (100)	425 (100)	
Serology	4 (0.6)	4 (1.6)	0 (0)	0 (0)	0 (0)		86 (3.4)	86 (4.4)	0 (0)	0 (0)	0 (0)	
Antigen	20 (3.1)	7 (2.8)	13 (26)	0 (0)	0 (0)		402 (16)	385 (19.8)	17 (32.1)	0 (0)	0 (0)	
COVID+ by >1 testing type	120 (18.4)	115 (46.2)	5 (10)	0 (0)	0 (0)		479 (19.1)	475 (24.4)	4 (7.5)	0 (0)	0 (0)	
SARS-CoV-2 (+), *n* (%)	652 (100)	249 (100)	50 (100)	23(100)	330 (100)	1	2505 (100)	1944 (100)	53 (100)	83 (100)	425 (100)	1
SARS-CoV-2 vaccination, *n* (%)						**0.02**						**0.005**
Yes	533 (84.7)	211 (84.7)	44 (88)	N/A	278 (84.2)		2149 (88.7)	1719 (88.4)	51 (96.2)	N/A	379 (89.2)	
No	89 (14.1)	31 (12.4)	6 (12)	N/A	52 (15.8)		225 (9.3)	177 (9.1)	2 (3.8)	N/A	46 (10.8)	
Unknown	30 (4.6)	7 (2.8)	0 (0)	23 (100)	0 (0)		131 (5.4)	48 (2.5)	0 (0)	83 (100)	0 (0)	

*p*-values that are significant *p* < 0.05 are highlighted in bold.

Prevalence of pre-existing comorbidities in PNP and NNP patients is presented in Table 3. We have previously shown that the frequency of different comorbidities varies significantly between PNP and NNP patients. In this study, we further characterized comorbidities across four geographical areas. There were significant country-related differences in most pre-existing comorbidities.

**Table 3:** Comorbidities in post-hospitalization and non-hospitalized Neuro-PASC patients in the United States, Colombia, Nigeria, and India.

	Overall PNP	USA PNP	Colombia PNP	Nigeria PNP	India PNP	*p-*value	Overall NNP	USA NNP	Colombia NNP	Nigeria NNP	India NNP	*p-*value
*n*	652	249	50	23	330		2505	1944	53	83	425	
Pre-existing comorbidity *n* (%)												
Hypertension	170 (26.1)	106 (42.6)	16 (32)	10 (43.5)	38 (11.5)	**<0.0001**	419 (16.7)	355 (18.3)	7 (13.2)	12 (14.6)	45 (10.6)	**0.0014**
Headache	125 (19.9)	28 (11.2)	5 (10)	n/a	92 (27.9)	**<0.0001**	456 (18.8)	363 (18.7)	5 (9.4)	n/a	88 (20.7)	0.1
Depression/anxiety	116 (17.8)	61 (24.5)	4 (8)	n/a	51 (15.5)	**0.0003**	699 (27.9)	640 (32.9)	10 (20.8)	n/a	49 (11.5)	**0.0003**
Type 2 diabetes	111 (17)	57 (22.9)	9 (18)	5 (21.7)	40 (12.1)	**0.007**	154 (6.1)	116 (6.0)	1 (1.9)	6 (7.2)	31 (7.3)	0.4
Dyslipidemia	108 (16.6)	57 (22.9)	12 (24)	0 (0)	39 (11.8)	**0.0004**	365 (14.6)	324 (16.7)	6 (11.3)	0 (0)	35 (8)	**<0.0001**
Other endocrine disorders	88 (13.5)	80 (32.1)	6 (12)	2 (8.7)	0 (0)	**0.001**	413 (16.5)	408 (21.0)	5 (9.4)	0 (0)	0 (0)	**<0.0001**
Lung disease	85 (13)	71 (28.5)	6 (12)	0 (0)	8 (2.4)	**<0.0001**	375 (15)	365 (18.8)	1 (1.9)	0 (0)	9 (2.1)	**<0.0001**
Gastrointestinal disease	65 (10)	60 (24.1)	3 (6.0)	2 (8.7)	0 (0)	**0.003**	342 (13.7)	342 (17.6)	0 (0)	0 (0)	0 (0)	**<0.0001**
Insomnia	57 (8.9)	16 (6.4)	0 (0)	n/a	40 (12.1)	**0.003**	334 (13.8)	194 (10.0)	4 (7.5)	n/a	136 (32)	**<0.0001**
Cardiovascular disease	50 (7.7)	43 (17.3)	6 (12)	1 (4.3)	0 (0)	**<0.0001**	177 (7.1)	177 (9.1)	0 (0)	0 (0)	0 (0)	**<0.0001**
Autoimmune disease	41 (6.3)	31 (12.4)	6 (12)	0 (0)	4 (1.2)	**<0.0001**	138 (11.1)	131 (6.7)	7 (13.2)	0 (0)	0 (0)	**<0.0001**
Neuropsychiatric disease	36 (5.5)	36 (14.5)	0 (0)	0 (0)	0 (0)	**<0.0001**	325 (13)	316 (16.3)	0 (0)	0 (0)	9 (0)	**<0.0001**
Cancer	30 (4.6)	29 (11.6)	1 (2)	0 (0)	0 (0)	**<0.0001**	124 (5.0)	124 (6.4)	0 (0)	0 (0)	0 (0)	**<0.0001**
Chronic kidney disease	13 (2)	10 (4.0)	0 (0)	0 (0)	3 (0.9)	**0.001**	41 (1.6)	37 (1.9)	0 (0)	0 (0)	4 (1.6)	0.4
Peripheral vascular disease	13 (2)	13 (4.8)	0 (0)	0 (0)	0 (0)	**0.0001**	53 (2.1)	52 (2.7)	1 (1.9)	0 (0)	0 (0)	**0.0003**
Cerebrovascular disease	7 (1.1)	6 (2.4)	1 (4.3)	n/a	0 (0)	**<0.0001**	17 (0.7)	16 (0.8)	1 (1.9)	n/a	0 (0)	**<0.0001**
Neuromuscular disease	6 (0.9)	6 (2.2)	0 (0)	0 (0)	0 (0)	**0.02**	16 (0.6)	16 (0.8)	0 (0)	0 (0)	0 (0)	0.3
Dysautonomia	5 (0.8)	4 (1.6)	1 (2)	n/a	0 (0)	**0.03**	42 (1.7)	38 (2.0)	4 (7.5)	n/a	0 (0)	**<0.0001**
Traumatic brain injury	5 (0.8)	5 (1.8)	0 (0)	0 (0)	0 (0)	**0.05**	79 (3.2)	79 (4.1)	0 (0)	0 (0)	0 (0)	**<0.0001**
Organ transplant	0 (0)	0 (0)	0 (0)	0 (0)	0 (0)	1	8 (0.3)	7 (0.4)	0 (0)	1 (1.2)	0 (0)	0.3
Other	30 (4.6)	23 (9.2)	3 (6)	4 (17.4)	0 (0)	0.3	317 (12.7)	314 (16.2)	1 (1.9)	2 (2.4)	0 (0)	**<0.0001**

*p*-values that are significant *p* < 0.05 are highlighted in bold.

### Outcome data

#### Differences in neuro-PASC and other symptoms frequency between countries

Table 4 shows Neurologic symptoms and signs attributed to Long COVID. Overall, PNP patients were evaluated 24.6 months after symptom onset, with a range from 15.6 months (USA) to 31.2 months (India) and NNP patients overall were seen 19.3 months after symptom onset, with a range of 17.6 months (USA) and 27 months (India). Across both PNP and NNP cohorts, the most consistently reported symptoms were brain fog, myalgia, dizziness, headache, pain other than chest, numbness/tingling, anosmia, dysgeusia, tinnitus and blurry vision, with significant differences between the four geographic locations for all of them (*p* < 0.0001). There were significant differences in the median number of neurologic manifestations attributed to PASC in both PNP and NNP patients, ranging from 5 in the USA to 1 in India. Among PNP patients, brain fog was reported by 87.1% in the USA, 56% in Colombia, 47.8% in Nigeria, and only 12.1% in India (*p* < 0.0001). Using Colombian PNP patients as a reference, brain fog was less often reported in India [OR 0.09 (95% CI 0.05, 0.18), *p* < 0.0001], more often reported in the USA [OR 4.66 (95% CI 2.31, 9.39), *p* < 0.0001], and reported at similar rate in Nigeria [OR 0.55 (95% CI 0.20, 1.51), *p* = 0.25] after adjusting for age (*p* = 0.33) and sex (*p* = 0.71). Similar patterns emerged in the NNP group, where brain fog affected 86% of USA. patients, 62.3% in Colombia, 62.7% in Nigeria, and just 14.6% in India (*p* < 0.0001). Using Colombian NNP patients as a reference, brain fog was less often reported in India [OR 0.07 (95% CI 0.03, 0.13), *p* < 0.0001], more often reported in the USA [OR 2.29 (95% CI 1.22, 4.28), *p* = 0.010], and reported at similar rate in Nigeria [OR 0.65 (95% CI 0.30, 1.38), *p* = 0.26] after adjusting for age (*p* = 0.57) and sex [male OR 0.77 (95% CI 0.61, 0.97), *p* = 0.02]. Myalgia was also highly prevalent among PNP patients, reported by 55% in the U.S., 52% in Colombia, 31.2% in India, but only 8.7% in Nigeria (*p* < 0.0001), with similar trends observed in NNP patients: 53.7% in the U.S., 32.1% in Colombia, 23.1% in India, and only 9.6% in Nigeria (*p* < 0.0001). Dizziness followed the same trajectory, affecting 56.6% of PNP patients in the U.S., 28% in Colombia, 32.1% in India and 8.7% in Nigeria, (*p* < 0.0001); in NNPs, dizziness was reported in 58.3% of U.S. patients, 32.1% in Colombia, 19.3% in India and only 7.2% in Nigeria (*p* < 0.0001). These findings highlight differences in the frequency of neurologic manifestations of PASC based on the geographical location of the patients. Specifically, the pattern of neurologic manifestations of PASC differed between North and South America, Africa and Asia.

**Table 4:** Neurologic symptoms and signs attributed to Long COVID in post-hospitalization and non-hospitalized Neuro-PASC patients in the United States, Colombia, Nigeria, and India.

	Overall PNP	USA PNP	Colombia PNP	Nigeria PNP	India PNP	*p*-value	Overall NNP	USA NNP	Colombia NNP	Nigeria NNP	India NNP	*p*-value
*n*	652	249	50	23	330		2,505	1944	53	83	425	
Time from symptom onset to clinic visit (month, mean (1 SD))	24.6 (10.4)	15.6 (11.3)	26.3 (7.3)	22.8 (1.7)	31.2 (2.4)	**<0.0001**	19.3 (26)	17.6 (29.1)	24.7 (9.3)	16.6 (5.7)	27 (2.8)	**<0.0001**
Neurologic manifestations or symptoms attributed to PASC [median (IQR)]	3 [1–5]	5 [3–7]	3.5 [2–6]	2 [2–3]	1 [1–3]	**<0.0001**	4 [2–6]	5 [3–7]	3 [2–4]	2 [2–3]	1 [1–2]	**<0.0001**
*n* (%)												
≥4	266 (40.8)	181 (72.7)	25 (50)	4 (17.4)	56 (17)	**<0.0001**	1,483 (59.2)	1,399 (72)	15 (28.3)	20 (24.1)	49 (11.5)	**<0.0001**
Brain fog	300 (46)	221 (88.8)	28 (56)	11 (47.8)	40 (12.1)	**<0.0001**	1819 (72.6)	1,672 (86)	33 (62.3)	52 (62.7)	62 (14.6)	**<0.0001**
Myalgia	268 (41.1)	137 (55)	26 (52)	2 (8.7)	103 (31.2)	**<0.0001**	1,167 (46.6)	1,044 (53.7)	17 (32.1)	8 (9.6)	98 (23.1)	**<0.0001**
Dizziness	263 (40.3)	141 (56.6)	14 (28)	2 (8.7)	106 (32.1)	**<0.0001**	1,239 (49.5)	1,134 (58.3)	17 (32.1)	6 (7.2)	82 (19.3)	**<0.0001**
Headache	245 (37.6)	133 (53.4)	16 (32)	4 (17.4)	92 (27.9)	**<0.0001**	1,442 (57.6)	1,312 (67.5)	13 (24.5)	29 (34.9)	88 (20.7)	**<0.0001**
Pain other than chest	230 (35.3)	125 (50.2)	22 (44)	3 (13)	80 (24.2)	**<0.0001**	1,009 (40.3)	902 (46.4)	15 (28)	2 (2.4)	90 (21.2)	**<0.0001**
Numbness/tingling	214 (32.8)	149 (59.8)	29 (58)	8 (34.8)	28 (8.5)	**<0.0001**	968 (38.6)	915 (47.1)	12 (22.6)	4 (4.8)	37 (8.7)	**<0.0001**
Anosmia	183 (28.1)	95 (38.2)	11 (22)	1 (4.3)	76 (23)	**<0.0001**	833 (33.3)	742 (38.2)	16 (30.2)	7 (8.4)	68 (16)	**<0.0001**
Dysgeusia	14 (21.8)	104 (41.8)	12 (24)	2 (8.7)	24 (7.3)	**<0.0001**	740 (29.5)	706 (36.3)	13 (25)	5 (6)	16 (3.8)	**<0.0001**
Tinnitus	144 (22.1)	92 (36.9)	16 (32)	0 (0)	36 (10.9)	**<0.0001**	816 (32.6)	781 (40.2)	10 (18.9)	1 (1.2)	24 (5.7)	**<0.0001**
Blurred vision	100 (15.3)	79 (31.7)	8 (16)	1 (4.3)	12 (3.6)	**<0.0001**	690 (27.5)	673 (34.6)	10 (18.9)	2 (2.4)	5 (1.2)	**<0.0001**
Ischemic stroke	8 (2.5)	6 (2.4)	2 (4)	0 (0)	n/a	0.80	27 (1.3)	27 (1.4)	0 (0)	0 (0)	n/a	0.81
Focal sensory deficit	6 (2)	1 (0.4)	5 (10)	n/a	n/a	**0.0006**	3 (0.15)	3 (0.15)	0 (0)	n/a	n/a	1
Seizure	5 (1.6)	3 (1.2)	2 (3.8)	0 (0)	n/a	0.32	39 (1.9)	37 (1.9)	0 (0)	2 (2.4)	n/a	0.66
Movement disorder	5 (1.6)	2 (0.8)	3 (6)	0 (0)	n/a	1	10 (0.48)	9 (0.46)	1 (2)	0 (0)	n/a	0.27
Focal motor deficit	4 (1.3)	0 (0)	4 (8)	n/a	n/a	**0.0007**	1 (0.05)	1 (0.05)	0 (0)	n/a	n/a	1
Meningitis	1 (0.31)	1 (0.4)	0 (0)	0 (0)	n/a	1	3 (0.14)	3 (0.15)	0 (0)	0 (0)	n/a	1
Encephalitis	1 (0.31)	0 (0)	1 (2)	0 (0)	n/a	0.23	6 (0.29)	5 (0.26)	0 (0)	1 (1.2)	n/a	0.33
Hemorrhagic stroke	1 (0.31)	0 (0)	1 (2)	0 (0)	n/a	0.23	1 (0.05)	1 (0.05)	0 (0)	0 (0)	n/a	1
Polyradiculitis	1 (0.31)	0 (0)	1 (2)	0 (0)	n/a	0.23	3 (0.14)	3 (0.15)	0 (0)	0 (0)	n/a	1
Other symptom, *n* (%)												
Fatigue	296 (45.4)	218 (57.6)	37 (74)	10 (43.5)	31 (9.4)	**<0.0001**	1967 (78.5)	1749 (90)	38 (71.7)	49 (59)	131 (30.8)	**<0.0001**
Depression/anxiety	265 (40.6)	173 (69.5)	34 (68)	2 (8.7)	56 (17)	**<0.0001**	1,531 (61.1)	1,433 (73.7)	21 (39.6)	5 (6.0)	72 (17)	**<0.0001**
Shortness of breath	252 (38.7)	172 (69.1)	27 (54)	3 (13)	50 (15.2)	**<0.0001**	945 (37.7)	916 (47.1)	11 (20.8)	3 (3.6)	15 (3.5)	**<0.0001**
Insomnia	227 (34.8)	158 (63.5)	23 (46)	6 (26.1)	40 (12.1)	**<0.0001**	1,285 (51.3)	1,136 (58.4)	17 (32.1)	28 (33.7)	104 (24.5)	**<0.0001**
Chest pain	127 (19.5)	97 (39)	11 (22)	4 (17.4)	15 (4.5)	**<0.0001**	655 (26.1)	601 (30.9)	5 (9.4)	3 (3.6)	46 (10.8)	**<0.0001**
Dysautonomia	91 (14)	86 (34.5)	4 (8)	1 (4.3)	0 (0)	**<0.0001**	745 (29.7)	737 (37.9)	7 (13.2)	1 (1.2)	0 (0)	**<0.0001**
GI symptoms	85 (13)	62 (24.9)	8 (16)	0 (0)	15 (4.5)	**<0.0001**	749 (29.9)	696 (35.8)	9 (17)	0 (0)	44 (10.4)	**<0.0001**
Sign *n* tested/total (%)												
Abnormal exam	188 (28.8)	122 (48.9)	32 (64)	11 (47.8)	33 (10)	**<0.0001**	958 (38.2)	867 (44.6)	23 (43.4)	18 (21.7)	50 (11.7)	**<0.0001**
Short-term memory deficits	117 (17.9)	79 (31.7)	20 (40)	8 (34.8)	10 (3.0)	**<0.0001**	685 (27.3)	606 (31.2)	15 (28.3)	44 (53)	20 (4.7)	**<0.0001**
Attention deficit	49 (16.4)	35 (14.1)	14 (28)	n/a	n/a	**0.02**	242 (12.1)	228 (11.7)	14 (26.4)	n/a	n/a	**0.004**
Sensory dysfunction	78 (11.9)	45 (18.1)	12 (24)	0 (0)	21 (6.4)	**<0.0001**	231 (9.2)	216 (11.1)	3 (5.7)	0 (0)	12 (2.8)	**<0.0001**
Gait dysfunction	72 (11.4)	39 (15.6)	11 (22)	n/a	22 (6.7)	**0.0001**	166 (6.9)	158 (8.1)	0 (0)	n/a	8 (1.9)	**<0.0001**
Motor dysfunction	56 (8.6)	25 (10)	7 (14)	0 (0)	24 (7.3)	0.15	91 (3.6)	85 (4.4)	0 (0)	0 (0)	6 (1.4)	**0.002**
Cranial nerve dysfunction	14 (4.7)	11 (4.4)	3 (6)	n/a	n/a	0.71	74 (3.7)	74 (3.8)	0 (0)	n/a	n/a	0.26
Cerebellar dysfunction	12 (0.5)	12 (4.8)	0 (0)	n/a	n/a	0.22	58 (2.9)	58 (3)	0 (0)	n/a	n/a	0.40
Movement disorder	11 (1.7)	4 (0.8)	3 (6)	0 (0)	4 (1.2)	0.15	23 (0.92)	18 (0.9)	1 (1.9)	0 (0)	4 (0.9)	0.60

*p*-values that are significant *p* < 0.05 are highlighted in bold.

The main non-neurologic symptoms of PASC overall included fatigue, depression/anxiety, shortness of breath and insomnia, with significant differences between the four geographic locations for all of them (*p* < 0.0001). Interestingly, depression/anxiety was reported among PNP patients in 69.5% in USA, 68% in Colombia, compared to only 8.7% in Nigeria and 17% in India. Using Colombian PNP patients as a reference, depression/anxiety was less often reported in India [OR 0.07 (95% CI 0.03, 0.14), *p* < 0.0001] and Nigeria [OR 0.03 (95% CI 0.01, 0.17), *p* < 0.0001] and reported at similar rate in the USA [OR 0.81 (95% CI 0.40, 1.62), *p* = 0.55] after adjusting for age (*p* = 0.61) and sex (*p* = 0.44). Similarly, among NNP patients, depression/anxiety was reported in 73% in USA, 39.6% in Colombia, compared to only 6% in Nigeria and 17% in India. Using Colombian NNP patients at a reference, depression/anxiety was less often reported in India [OR 0.09 (95% CI 0.05, 0.17), *p* < 0.0001] and Nigeria [OR 0.03 (95% CI 0.01, 0.08), *p* < 0.0001] and reported at similar rate in the USA [OR 1.24 (95% CI 0.68, 2.25), *p* = 0.48] after adjusting for age (*p* = 0.36) and sex (*p* = 0.37).

#### Multiple correspondence analysis

**Figure 2:**
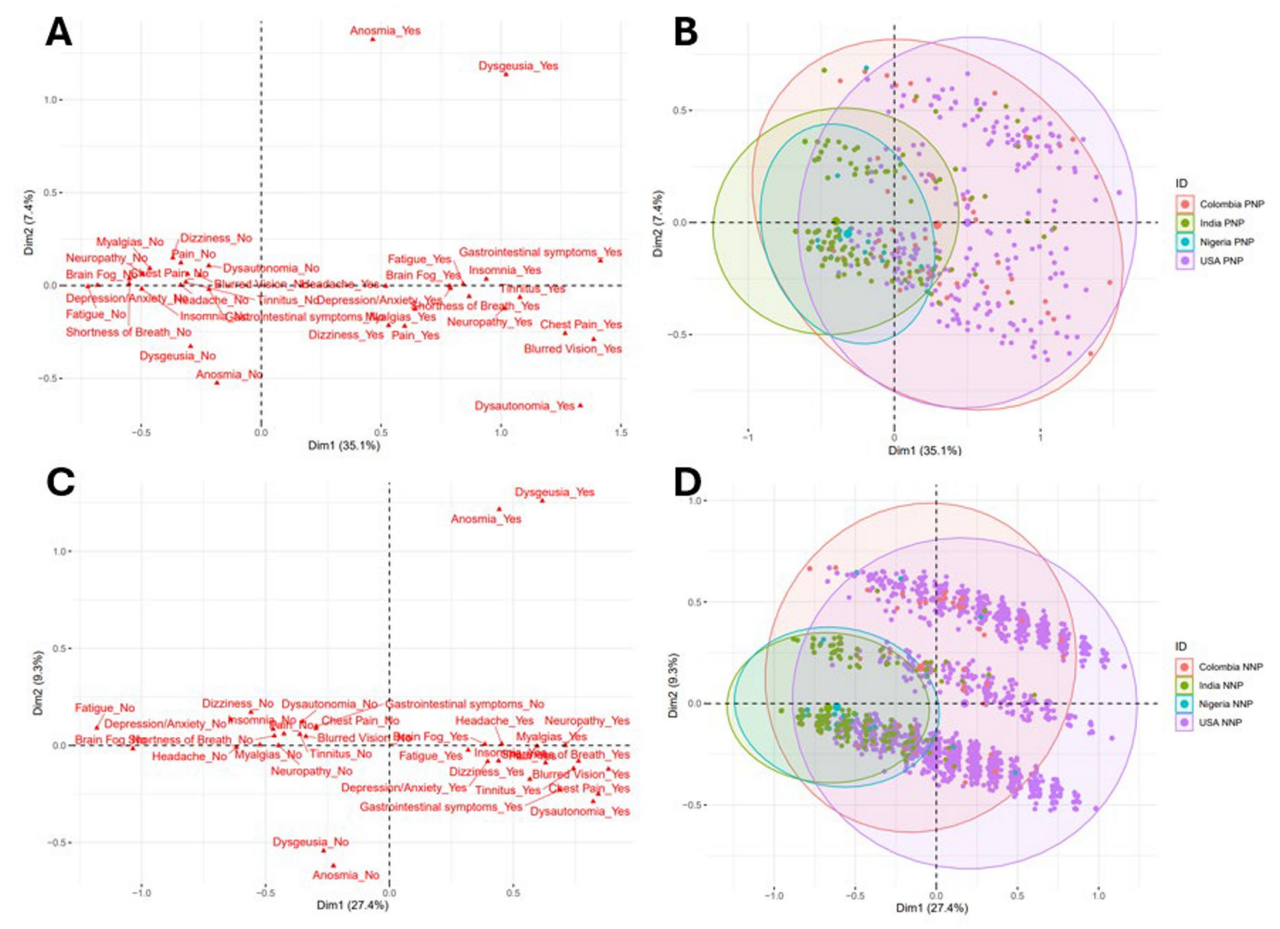
Multiple correspondence analysis of Neuro-PASC symptoms by country. **(A)** PNP cohort symptom point cloud. **(B)** PNP cohort patient point cloud with concentration ellipses by country. **(C)** NNP cohort symptom point cloud. **(D)** NNP cohort patient point cloud with concentration ellipses by country. Larger-sized points represent the mean values of each respective country’s distribution. Increasing distance between the origin and a given symptom category indicates a greater contribution of that category to the pole of the corresponding dimension. Symptom categories with similar profiles of patients are grouped together. For both PNP and NNP, dimension 1 globally separates “yes” from “no” symptom categories such that “yes” symptom categories with larger dimension 1 values are toward the right of the graph. For both PNP and NNP, the presence or absence of anosmia and dysgeusia are the predominant contributors to dimension 2. The predominance of anosmia and dysgeusia in dimension 2, relative to other symptoms, results in a visually stratified patient symptom cloud (particularly for the NNP cohort) such that presence of anosmia and dysgeusia with larger dimension 2 values are toward the top of the graph. For both PNP and NNP cohorts, there was a significant difference in dimension 1 between countries (*p* < 0.001) but no significant difference in dimension 2 (*p* = 0.31 and *p* = 0.13, respectively). For both PNP and NNP cohorts, the US and Colombia symptom profiles were more similar while India’s symptom profile was more similar to Nigeria’s. The larger dimension 1 values for the U.S. and Colombia suggest a more severe global symptom profile with more frequent “yes” symptom responses than India and Nigeria.

Seventeen symptoms were reported as present in ≥ 10% of patients and were therefore included in the MCAs, graphically displayed in Figure 2. For the PNP cohort, dimension 1 explained 35.1% of the variance while dimension 2 explained 7.4% of the variance; each of the remaining fifteen dimensions explained ≤ 6.2% of the variance. Thirteen symptoms had correlation coefficient squared values (r^2^) with PNP dimension 1 that reached 0.25: fatigue (0.61), brain fog (0.54), neuropathy (0.50), shortness of breath (0.48), insomnia (0.47), depression/anxiety (0.43), chest pain (0.39), blurred vision (0.36) tinnitus (0.34), gastrointestinal symptoms (0.31), dysgeusia (0.30), myalgias (0.30), and dysautonomia (0.29) with all other symptoms having r^2^ values between 0.09 and 0.20. For PNP dimension 2, only two symptoms had r^2^ values exceeding 0.25: anosmia (0.69) and dysgeusia (0.37), with all other symptoms having r^2^ values below 0.07. MCA results for PNP patients are displayed in Figures 2A,B.

For the NNP cohort, dimension 1 explained 27.4% of the variance while dimension 2 explained 9.3% of the variance; each of the remaining fifteen dimensions explained < 7.0% of the variance. Thirteen symptoms had r^2^ values with NNP dimension 1 that reached 0.25: brain fog (0.40), fatigue (0.37), shortness of breath (0.35), dizziness (0.32), neuropathy (0.32) myalgias (0.31), blurred vision (0.30), dysautonomia (0.29), headache (0.28), pain (0.27), tinnitus (0.27), depression/anxiety (0.25), chest pain (0.25) with all other symptoms having r^2^ values between 0.10 and 0.21. For NNP dimension 2, only two symptoms had r^2^ values exceeding 0.25: anosmia (0.75) and dysgeusia (0.68), with all other symptoms having r^2^ values below 0.04. MCA results for NNP patients are displayed in Figures 2C,D.

For both PNP [median (IQR): Columbia 0.203 (−0.049, 0.614), India −0.544 (−0.590, −0.337), Nigeria −0.374 (−0.434, −0.196), US 0.501 (0.137, 0.890)] and NNP [median (IQR): Columbia −0.102 (−0.386, 0.165), India −0.726 (−0.836, −0.564), Nigeria −0.666 (−0.795, −0.507), US 0.171 (−0.143, 0.489)] there was a significant difference in dimension 1 between countries (*p* < 0.001), with the US being more similar to Colombia and India being more similar to Nigeria. For both PNP and NNP patients, dimension 1 globally separates “yes” from “no” symptom categories such that “yes” symptom categories have larger dimension 1 values. Using Colombian PNP patients as a reference, dimension 1 was lower in India [*β* = −0.71 (95% CI −0.83, −0.59), *p* < 0.0001] and Nigeria [*p* = −0.60 (95% CI −0.79, −0.40), *p* < 0.0001] and higher in the USA *p* = 0.21 (95% CI 0.08, 0.33), *p* = 0.001 after adjusting for age (*p* = 0.18) and sex [male *p* = −0.08 (95% CI −0.14, −0.02), *p* = 0.008]. Using Colombian NNP patients as a reference, dimension 1 was lower in India [*p* = −0.54 (95% CI −0.65, −0.42), *p* < 0.0001] and Nigeria [*p* = −0.49 (95% CI −0.63, −0.35), *p* < 0.0001] and higher in the USA [*p* = 0.28 (95% CI 0.17, 0.39), *p* < 0.0001] after adjusting for age (*p* = 0.22) and sex [male *p* = −0.10 (95% CI −0.13, −0.07), *p* < 0.0001]. Therefore, the larger dimension 1 values for the US and Colombia suggest a more severe global symptom profile with more frequent “yes” symptom responses than India and Nigeria. Dimension 2 values were not significantly different between countries for either PNP or NNP (*p* = 0.31 and *p* = 0.13, respectively).

#### Alterations in the neurological exam

Overall, 28.8% of PNP patients had an abnormal neurologic exam, with 48.9% in the U.S., 64% in Colombia, and 47.8% in Nigeria, compared to only 10% in India (*p* < 0.0001). Similarly, 38.2% of NNP patients overall had an abnormal neurological exam, including 44.6% in the U.S., 43.4% in Colombia, 21.7% in Nigeria, and only 11.7% in India (*p* < 0.0001). Across both PNP and NNP groups, the most commonly reported signs were short-term memory deficits and sensory dysfunction, suggesting a persistent pattern of cognitive and sensory impairment regardless of hospitalization status.

#### Quality of life measures

**Figure 3:**
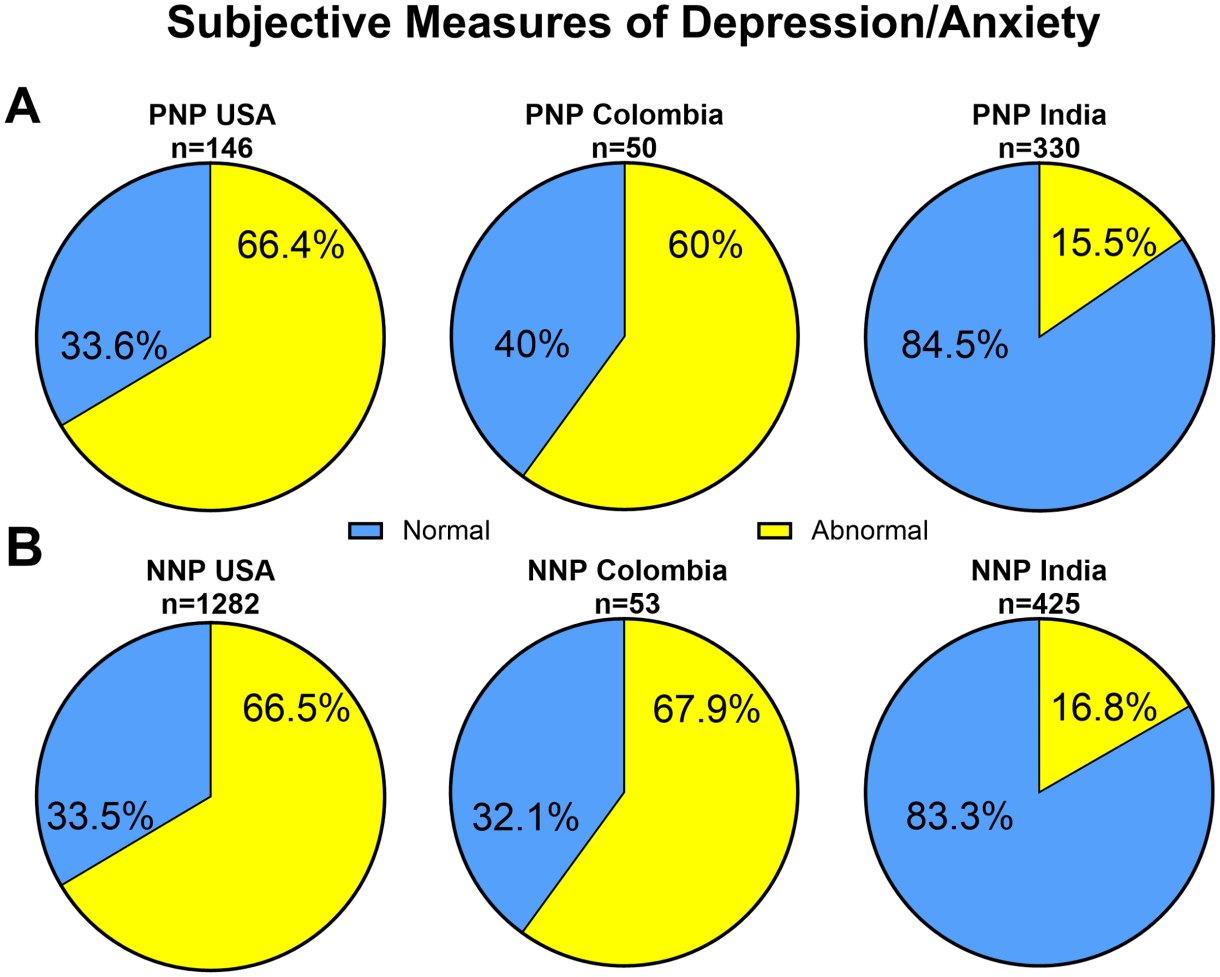
Pie charts illustrating the proportion of **(A)** PNP and **(B)** NNP patients classified as having normal versus abnormal subjective measures of depression and anxiety in the USA (PROMIS), Colombia (PROMIS) and India (DASS-21). Measure status was determined using standardized test cutoffs for anxiety and depression. Different assessment tools were used in USA/Colombia and India, and direct comparison of impairment rates should be interpreted with caution.

We analyzed the subjective quality of life (QoL) in the domains of depression and anxiety, measured using composite scores of standardized PROMIS and DASS-21 tools that were available from the US, Colombia and India at the time of the study visit. These revealed significant differences across regions (Figure 3). Among PNP patients, 66.4% in the US and 60% in Colombia had alteration of QoL in the domains of depression and anxiety, compared to only 15.5% of Indian patients (*p* < 0.0001). Similarly, among NNP patients, 66.5% in the US and 67.9% in Colombia had alteration of QoL in the domains of depression and anxiety, compared to only 16.8% of Indian patients (*p* < 0.0001).

#### Cognitive outcomes

**Figure 4:**
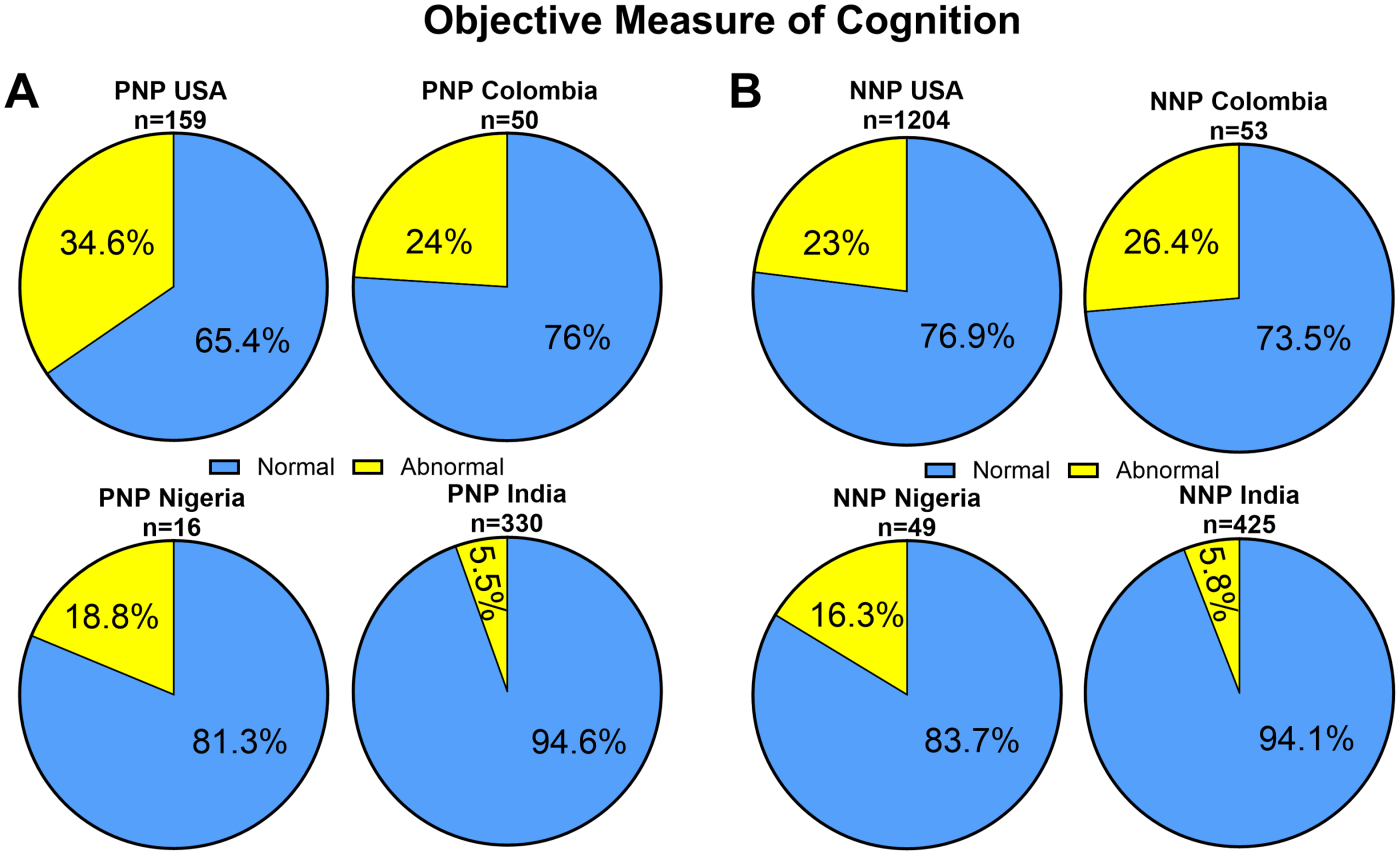
Pie charts illustrating the proportion **(A)** PNP and **(B)** NNP patients classified as having normal versus abnormal objectives measures of cognition in the USA (NIH Toolbox), Colombia (NIH Toolbox), Nigeria (MoCa), and India (MMSE). Cognitive status was determined using standardized test cutoffs. Different assessment tools were used in USA/Colombia, Nigeria and India and direct comparison of impairment rates should be interpreted with caution.

We then compared objective outcomes of cognitive function across regions, which was assessed using composite scores of the NIH Toolbox in the U.S. and Colombia, the Montreal Cognitive Assessment (MoCA) in Nigeria, and the Mini-Mental State Examination (MMSE) in India. These revealed significant geographic disparities in cognitive impairment among Neuro-PASC patients (Figure 4). Among PNP participants, 34.6% of patients in the U.S. exhibited abnormal cognitive scores, followed by 24% in Colombia and 18.4.4% in Nigeria, and 5.5% in India (*p* < 0.0001). A similar pattern emerged in the NNP group: 23%% in the U.S., 26.4% in Colombia, and 16.3% in Nigeria and 5.8% in India were classified as having impaired cognition (*p* < 0.0001).

## Discussion

This study aimed at comparing demographics, comorbidities, neurologic symptoms of Long COVID, patient-reported outcomes and cognitive findings in Neuro-PASC patients in the U.S., Colombia, Nigeria and India. As in our previous studies, we analyzed PNP and NNP groups separately. To the best of our knowledge, this is the first study to objectively compare neurologic manifestations of Long COVID in North America, Latin America, Africa and India, including countries with diverse income. The most frequent neurologic symptoms were brain fog, myalgia, dizziness, headache, and sensory disturbances, with frequency highest in the U.S. and lowest in India. There were significant differences for most neurologic and non-neurologic symptoms of PASC, driven by higher frequencies in U.S. and Colombia in both PNP and NNP cohorts. Our findings add to the body of knowledge in Global Neurology pertaining to Long COVID and highlight similarities and differences between various geographic areas. In particular, our findings indicate the larger number of NNP over PNP patients in each location, consistent with the evolution of the pandemic toward lesser severity of COVID-19 and outpatient management of most cases.

However, our study also shows important differences in demographics, especially in the gender composition of the patients with both PNP and NNP groups having a predominance of females in the U.S. and males in India. We and others have observed consistently that females more frequently than males consult at post-COVID clinics in the U.S. and Colombia (Bailey et al., 2023; Choudhury et al., 2024; Hurtado et al., 2024; Shah et al., 2025), consistent with the hypothesis that Long COVID is a new auto-immune disorder and the evidence that women are more prone than men to developing auto-immune diseases such as multiple sclerosis, rheumatoid arthritis or disseminated Lupus (Duquette et al., 1992; Myasoedova et al., 2010; Chakravarty et al., 2007). Conversely, there are significant gender disparities in health care utilization in India, with women facing barriers in accessing and receiving care compared to men, which likely explain the lower frequency of Indian women in our study (Dupas and Jain, 2024).

While the top 10 neurologic manifestations of Long COVID were similar across countries, the neurologic symptoms burden and respective frequencies of each of them differed significantly. Specifically, U.S. patients had the highest number of neurologic manifestations attributed to PASC in both PNP and NNP groups, followed by Colombia, Nigeria and India. In addition, both PNP and NNP Indian patients complained much less frequently of brain fog than those from other countries. Similarly, both Nigerian and Indian patients complained less frequently of depression/anxiety than those from the U.S. and Colombia in both PNP and NNP groups. The reluctance of disclosing cognitive and mental health issues in Nigeria and India is likely complex and multifactorial. These patterns are consistent with the possibility that cultural denial of mood disorder symptoms as well as a combination of stigma, misperceptions, religiosity and belief systems, and lack of health literacy may contribute to biased reporting (Gureje et al., 2010; Gaiha et al., 2020). This may be compounded by a dearth of mental health providers and perceived treatment options in those countries (Fadele et al., 2024).

Consequently, the MCA analysis confirms those differences and provides a very compelling visual representation of Long COVID symptom burden, with overlapping ellipses indicating a grouping of the U.S. and Colombia together on the one hand, and of Nigeria and India on the other, both among the PNP and NNP groups. Furthermore, this analysis showed that PNP and NNP patients from the U.S. and Colombia suffered from a higher symptom burden than those from Nigeria and India. One possibility is that these differences are caused by race and ethnicity, with the predominantly White (U.S.) and White Hispanic (in the region of Medellin in Colombia) population being more severely affected by Long COVID than the Black (Nigeria) and Asian (India) populations. Another possibility is that people were affected by different strains of SARS-CoV-2 in those geographic locations. Although no virologic data was collected for this study, this seems improbable as similar SARS-CoV-2 strain evolution has been occurring in parallel worldwide, since the beginning of the pandemic.

Sociocultural factors appear more likely to play a role in shaping how people relate to their Long COVID symptoms. Discussing mental health issues is routinely done during medical encounters in the U.S. and Colombia, while it is not customary in Nigeria or India (Fadele et al., 2024; Raghavan et al., 2023). This is consistent with the responses to the quality of life questionnaires, showing that close to two thirds of participants from the U.S. and Colombia expressed abnormal subjective impression of depression and anxiety in both PNP and NNP groups, while it was the case in only a sixth of Indian participants.

Previous studies have evaluated the outcomes of individuals with Long COVID across the globe. Our study is unique and adds to existing knowledge regarding the impact of neurological symptoms and their distribution globally while analyzing differences in geographic locations and country of different income categories. Indeed, most publications were meta-analyses of studies on all aspects of Long COVID (Ramonfaur et al., 2024; Global Burden of Disease Long CC et al., 2022; Chen et al., 2022; Pazukhina et al., 2024), while another reported specifically on mental health and sleep disorder (Seighali et al., 2024). One meta-analysis of 125 studies including over 4 million patients focused on long term neurological and cognitive impact of Long COVID (Elboraay et al., 2025). This study found that overall, cognitive and sleep disorders, headache and dizziness were the most frequent neurologic symptoms as well as fatigue. However, this study did not include any patients from Sub-Saharan Africa and did not make any comparisons based on geographic location or national income. While meta-analyses are informed by a large number of studies, they also reflect a high heterogeneity in definitions, methodologies, selection and sampling. Conversely, our approach was directed by the same group of investigators in the US, and was carried out separately with Colombian, Nigerian and Indian collaborators.

Our study has several limitations. The participants in the U.S. and India consulted a post-COVID clinic, while those from Colombia and Nigeria were contacted based on SARS-CoV-2 infection records for the purpose of a study. Therefore, there were differences in time from symptom onset between the four populations and could also lead to potential information bias due to differing clinical interview styles. Reported frequencies of cognitive impairment varied across regions; however, comparisons are limited by the use of non-harmonized assessment tools. Indeed, although we strived to harmonize data gathering and analysis, tests of subjective quality of life and objective cognitive testing differed in Nigeria and India compared to those used in the U.S. and Colombia, consistent with culturally adapted methodologies and commonly used practices in those countries. This limitation is inherent to Global Health research in general and to a new disease like Long COVID/Neuro-PASC in particular.

## Conclusion

Five years after the beginning of the pandemic, COVID-19 has now become endemic despite the availability of vaccines and boosters. Therefore, individuals affected by COVID-19 will continue to develop Long COVID. A Long COVID household pulse survey performed by the National Center of Health Statistics between June 2022 and September 2024 estimated that 17.9% of all adults ever had Long COVID and 5.3% currently suffered from Long COVID at the last time point, which corresponds to approximately 14 million adults in the U.S. Our study shows that Neuro-PASC exists all over the world and suggests that variations in subjective perception of symptom severity may be based on socio-cultural factors.

Specifically, willingness to discuss cognitive and mental health issues in high and high-middle income countries such as the U.S. and Colombia is associated with a higher symptom burden of Long COVID compared to low-middle income countries like Nigeria and India. This may also be associated with the relative importance allocated to those symptoms compared to the daily difficulties inherent to living in a resource-limited setting.

Our study underscores the challenge of comparing Neuro-PASC burden across diverse settings when using different assessment methods. Future multinational studies should prioritize common data elements and validated cross-cultural tools to truly understand the global epidemiology and sociocultural determinants of Neuro-PASC.

As the risk of Long COVID continues to increase after iterative episodes of COVID-19 (Babalola et al., 2025), Neuro-PASC will remain an important, and in many areas, underrecognized cause of alteration of QoL and cognitive dysfunction. Neuro-PASC affects young and middle-aged adults in their prime, causing significant detrimental impact on the workforce, productivity and innovation all over the world. Our findings highlight the need for culturally adapted screening and diagnosis of Neuro-PASC worldwide, and for the integration of post-COVID care in healthcare systems to enable long term follow-up and therapeutic interventions.

## Data Availability

The raw data supporting the conclusions of this article will be made available by the authors, without undue reservation.
